# Presurgical nasoalveolar moulding treatment in cleft lip and palate patients

**DOI:** 10.4103/0970-0358.57188

**Published:** 2009-10

**Authors:** Barry H. Grayson, Pradip R. Shetye

**Affiliations:** Institute of Reconstructive Plastic Surgery, New York University Langone Medical Center, New York, USA

**Keywords:** Cleft lip and palate, Nasoalveolar moulding, Presurgical orthopaedics

## Abstract

Presurgical infant orthopedics has been employed since 1950 as an adjunctive neonatal therapy for the correction of cleft lip and palate. Most of these therapies did not address deformity of the nasal cartilage in unilateral and bilateral cleft lip and palate as well as the deficiency of the columella tissue in infants with bilateral cleft. The nasolaveolar molding (NAM) technique a new approach to presurgical infant orthopedics developed by Grayson reduces the severity of the initial cleft alveolar and nasal deformity. This enables the surgeon and the patient to enjoy the benefits associated with repair of a cleft deformity that is minimal in severity. This paper will discuss the appliance design, clinical management and biomechanical principles of nasolaveolar molding therapy. Long term studies on NAM therapy indicate better lip and nasal form, reduced oronasal fistula and labial deformities, 60 % reduction in the need for secondary alveolar bone grafting. No effect on growth of midface in sagittal and vertical plane has been recorded up to the age of 18 yrs. With proper training and clinical skills NAM has demonstrated tremendous benefit to the cleft patients as well as to the surgeon performing the repair.

## INTRODUCTION

Presurgical infant orthopaedics has been used in the treatment of cleft lip and palate patients for centuries. The early techniques were focused on elastic retraction of the protruding premaxilla followed by stabilization after surgical repair. In 1689, Hoffmann demonstrated the use of facial binding to narrow the cleft and prevent postsurgical dehiscence.[1] A similar technique was shown by Desault in 1790 to retract the maxilla before surgical repair in patients with bilateral cleft repair.[1] In 1844, Hullihen stressed the importance of presurgical preparation of clefts using an adhesive tape binding.[2] Esmarch and Kowalzig used a bonnet and strapping to stabilize the premaxilla after surgical retraction.[3] In 1927, Brophy demonstrated the passing of a silver wire through both the ends of the cleft alveolus, and then progressively tightened the wire to approximate the ends of the alveolus before lip repair.[4]

The modern school of presurgical orthopaedic treatment in cleft lip and plate was started by McNeil in 1950.[5] He used a series of plates to actively mould the alveolar segments into the desired position. Burston, an orthodontist, further developed McNeil's technique and made it popular.[6] In 1975, Georgiade and Latham introduced a pin-retained active appliance to simultaneously retract the premaxilla and expand the posterior segments over a period of several days.[7] Hotz in 1987 described the use of a passive orthopaedic plate to slowly align the cleft segments.[8]

In 1993, Grayson *et al*,.[9] described a new technique to presurgically mould the alveolus, lip and nose in infants born with cleft lip and palate. The original research on neonatal moulding of the nasal cartilage was performed by Matsuo[10–12] using silicone tubes to mould the nostril. The nasoalveolar moulding appliance (NAM) consists of an intraoral moulding plate with nasal stents to mould the alveolar ridge and nasal cartilage concurrently. The objective of the presurgical NAM is to reduce the severity of the original cleft deformity and thereby enable the surgeon to achieve better repair of the alveolus, lip and nose. Use of the NAM technique has also eliminated surgical columella reconstruction and the resultant scar tissue in bilateral cleft lip and palate.[13] The nasoalveolar moulding technique has been shown to significantly improve the surgical outcome of the primary repair in cleft lip and palate patients compared to other techniques of presurgical orthopaedics.[14]

## IMPRESSION TECHNIQUE

**Figure 1:**
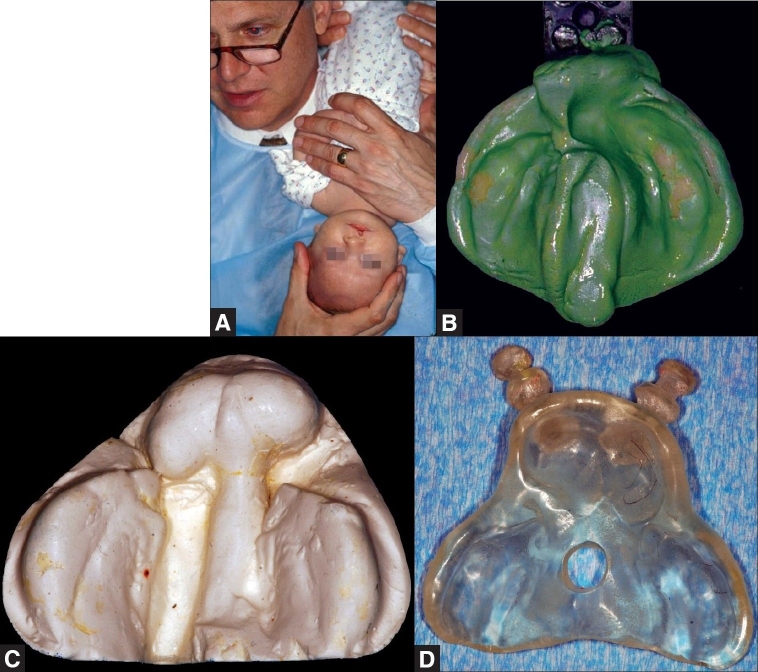
(A) Infant held in an inverted position during the impression process to prevent the tongue from falling back and to allow fluids to drain out. (B) Impression of a unilateral cleft patient using a custom tray and heavy-body silicone impression material. (C) Plaster stone working model of a bilateral cleft patient for appliance fabrication. (D) Bilateral nasoalveolar moulding plate with retention buttons fabricated using self-cure acrylic resin

Initial impression of the cleft lip and palate infant is obtained within the first week of birth. A heavy-bodied silicone impression material is used to take the initial impression. The impression can be taken in a clinical setting that is prepared to handle airway emergency, if at all encountered. A surgeon is always present during the impression process. The infant is held upside down by the surgeon and the impression tray is inserted into the oral cavity. The infant is held in an inverted position to prevent the tongue from falling back and to allow fluids to drain out of the oral cavity. The tray is seated until the impression material adequately covers the anatomy of the upper gum pads. Once the impression material is set, the tray is removed, and the mouth is examined for residual impression material. The impression is then poured with dental stone to obtain an accurate cast [Figure 1–C].

### Appliance fabrication and design

The moulding plate is fabricated on the dental stone model. All the undercuts and the cleft space are blocked with wax. The plate is made up of hard, clear self-cure acrylic and is trimmed with a denture soft material. The plate must be 2–3 mm in thickness to provide structural integrity and to permit adjustments during the process of moulding [Figure 1D]. The borders in the place of the frenum and other attachments must be adequately relived. A retention button is fabricated and positioned anteriorly at an angle of 40° to the plate. In the unilateral cleft only one retention arm is used. The exact location of the retention arm is determined at the chair side. It is positioned so as not to interfere with bringing the cleft lips together. The vertical position of the retention arm should be at the junction of the upper and lower lip. The retention button adequately secures the moulding plate in the mouth with the help of orthodontic elastics and tapes. A small opening measuring 6–8 mm in diameter is made on the palatal surface of the moulding plate to provide an airway in the event that the plate drops down posteriorly. The nasal stent is not fabricated at this time. Instead its construction is delayed until the cleft of the alveolus is reduced to about 5–6 mm in width.

### Appliance insertion and taping

**Figure 2:**
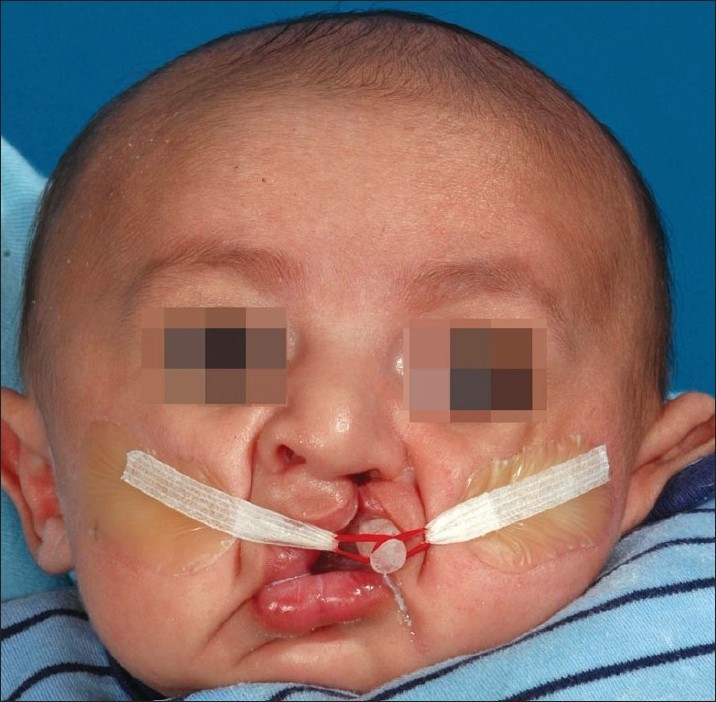
Unilateral cleft baby with a NAM plate showing the retention arm positioned approximately 40° down from the horizontal to achieve proper activation and to prevent unseating of the appliance from the palate. Note that there is no nasal stent placed for the first few weeks of treatment

The moulding plate is checked for overextension especially in the area of the vestibular folds as well as along the posterior border to check for any sharp edges or rough surfaces that may irritate the soft tissue. The appliance is then secured extraorally to the cheeks and bilaterally by surgical tapes that have orthodontic elastic bands at one end [Figure 2]. The use of skin barrier tapes on the cheeks like DuoDerm or Tegaderm is advocated to reduce irritation on the cheeks. The horizontal surgical tapes are a quarter inch in width and about 3–4 inches in length. The elastic on the surgical tape is looped on the retention arm of the moulding plate and the tape is secured to the cheeks. The elastics (inner diameter 0.25 inch, wall thickness heavy) should be stretched approximately two times their resting diameter for proper activation force of about 100 grams. The amount of force could vary depending on clinical objective and the mucosal tolerance to ulceration. Additional tapes may be necessary to secure the horizontal tape to the cheeks. Parents are instructed to keep the plate in the mouth full time and to remove it for daily cleaning. The infant may require time to adjust to feeding with the NAM appliance in the first few days.

### Appliance adjustments

The baby is seen weekly to make adjustments to the moulding plate to bring the alveolar segments together. These adjustments are made by selectively removing the hard acrylic and adding the soft denture base material to the moulding plate. No more than 1 mm of modification of the moulding plate should be made at one visit. The alveolar segments should be directed to its final and optimal position. Care must be taken to prevent the soft denture material from building up on the height of the alveolar crest as this will prevent complete seating of the moulding plate.

### Incorporation of the nasal stent

The nasal stent component of the NAM appliance is incorporated when the width of the alveolar gap is reduced to about 5 mm. The rationale for delaying the addition of the nasal stent is that as the alveolar gap is reduced, the base of the nose and the lip segment alignment is also improved. The alar rim, which at birth was stretched over a wide alveolar cleft deformity, will show some laxity, and with the nasal stent, this can be elevated into a symmetrical and convex form. The stent is made up of 0.36 inch, round stainless steel wire and takes the shape of a ‘Swan Neck’.[15] To appreciate the correct shape and orientation of the wire stent, one can use a roll of soft wax and make a template seated on the moulding plate. The stent is attached to the labial flange of the moulding plate, near the base of the retention arm. It extends forward and then curves backwards (in the form of a swan neck) entering 3–4 mm past the nostril aperture. As the wire extends into the nostril, it is curved back on itself to create a small loop for the retention of the intranasal portion of the nasal stent. The hard acrylic component is shaped into a bi-lobed form that resembles a kidney. A layer of soft denture liner is added to the hard acrylic for comfort. The upper lobe enters the nose and gently lifts forward the dome until a moderate amount of tissue blanching is evident. The lower lobe of the stent lifts the nostril apex and defines the top of the columella [Figure 3].

**Figure 3:**
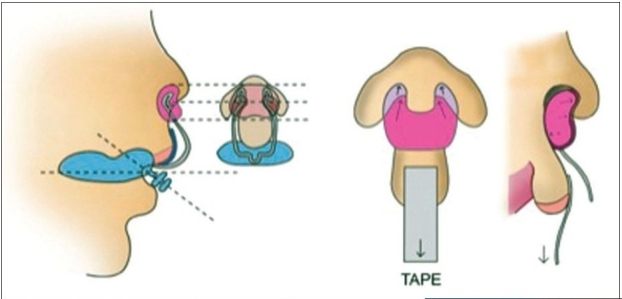
(A) Figure showing the design of the nasal stent and the position of the nasal stent in the nostril.

**Figure 3:**
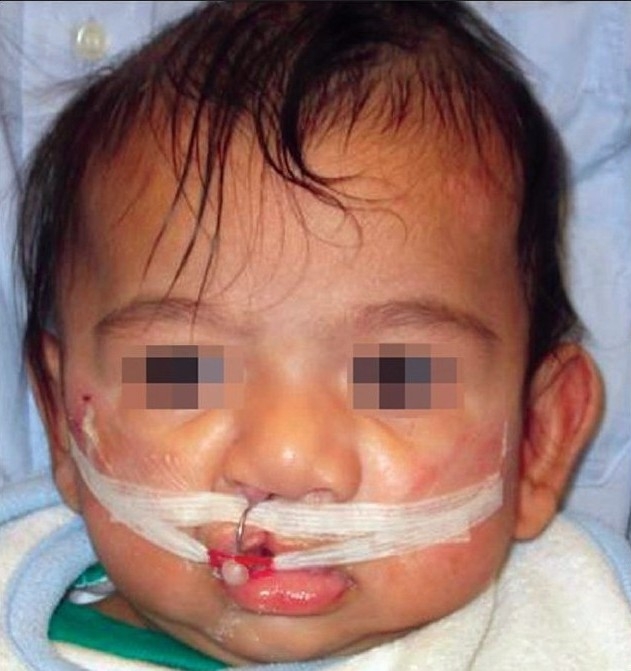
(B) Unilateral NAM plate with a nasal stent showing lip taping.

**Figure 3:**
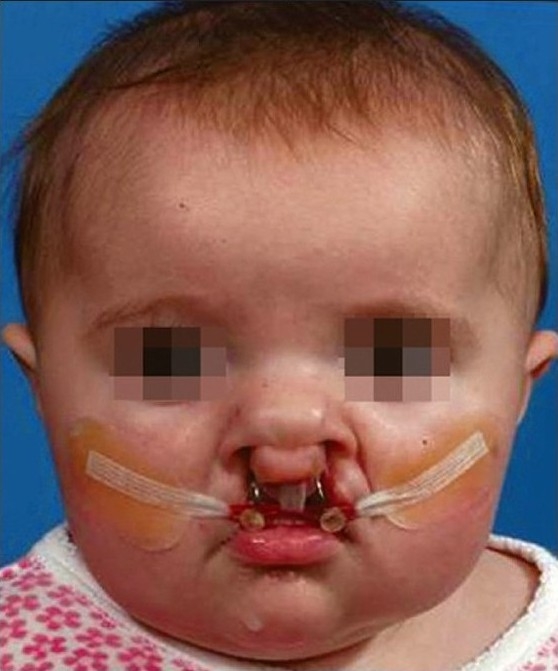
(C) The bilateral NAM plate in position showing the tape adhered to the prolabium and stretched to the plate and attached to the plate

### Non-surgical columella lengthening in bilateral cleft lip and palate

In bilateral cases, there is a need for two retention arms as well as two nasal stents which are similar in shape to the unilateral stent. After adding the nasal stents in the bilateral cleft, the attention is focused on non-surgical lengthening of the columella. To achieve this objective, a horizontal band of the denture material is added to join the left and right lower lobes of the nasal stent, spanning the base of the columella. This band sits at the nasolabial junction and defines this angle as the nasal tip continues to be lifted and projected forward. The tape is adhered to the prolabium underneath the horizontal lip tape and stretches downward to engage the retention arm with elastics. This vertical pull provides a counter stretch to the upward force applied to the nasal tip of the nasal stent. Taping downwards on the prolabium helps to lengthen the columella and vertically lengthens the often small prolabium. The horizontal lip tape is added after the prolabium tape is in place.

### Primary surgical repair of the alveolus, lip and nose

**Figure 4:**
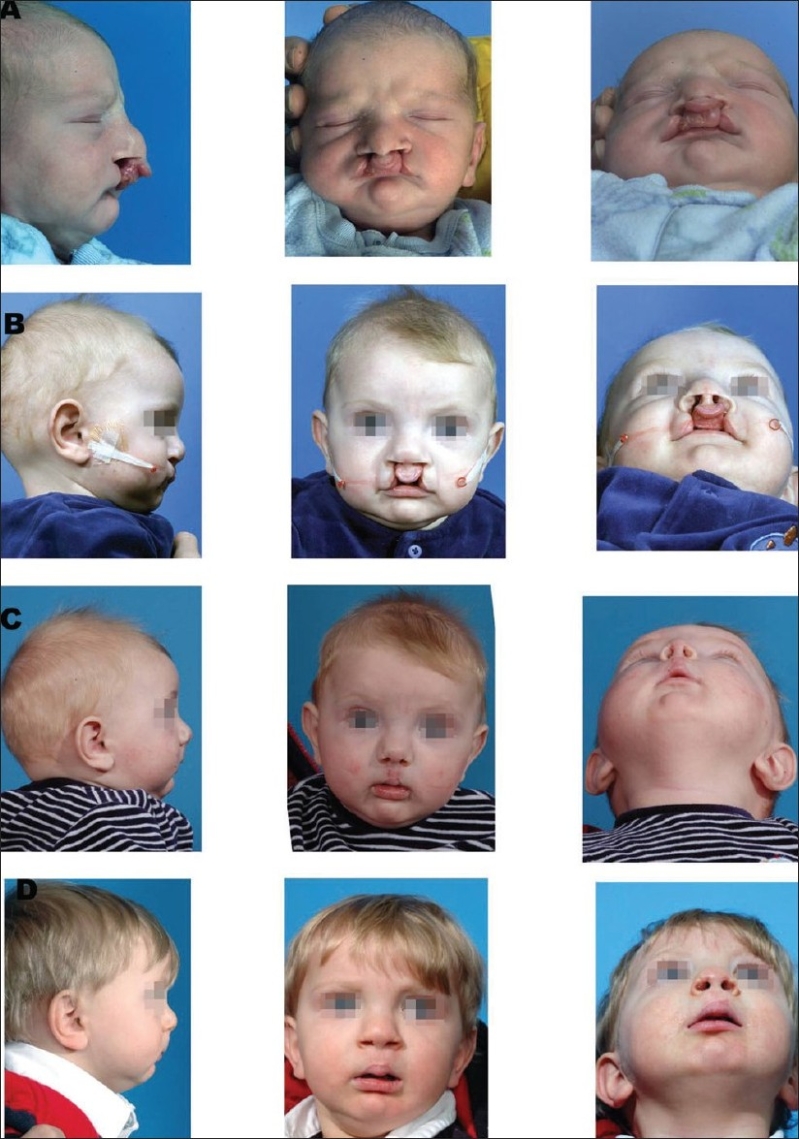
A bilateral complete cleft lip and palate infant treated with NAM therapy. (A) Prenasoalveolar moulding therapy, (B) post-nasoalveolar moulding therapy. Note the non-surgical columella elongation, (C) postsurgical photograph, (D) 1-year follow-up

The objective of NAM therapy is accomplished before the primary surgical repair. Surgical closure of the lip and nose is performed from 3–4 months of age.[16–18] Bilateral cleft patients tend to take one to two additional months to achieve presurgical clinical objectives. The duration of moulding therapy could also vary depending on the severity of the initial cleft deformity. The surgical technique must be modified to take advantage of the NAM preparation. The approximation of the alveolar segments permits the surgeon to perform gingivoperiosteoplasty. Reshaping of the deformed alar cartilage and stretching of the nasal mucosa enhances the surgeons' ability to achieve a good surgical repair. An appropriate modification of the surgical technique will improve the long-term retention of the surgical repair [Figure 4].

## COMPLICATIONS

The most common problems observed during NAM therapy are irritation to the oral mucosa, gingival tissue or nasal mucosa. Intraoral tissues may ulcerate from excessive pressure applied by the appliance. These are commonly found in the oral vestibule and on the labial side of the premaxilla. The oral and the nasal cavities of the infant should be carefully examined on each visit for ulceration and appropriate adjustments should be made to the moulding plate to relieve sore spots. The intranasal lining of the nasal tip can become inflamed if too much force is applied by the upper lobe of the nasal stent. The area under the horizontal prolabium band can become ulcerated if the band is too tight.

Another area of tissue irritation is the cheeks. Extreme care should be taken while removing the cheek tape to avoid any irritation to the skin. Skin barrier tapes like Tegaderm™ are recommended. Slight relocation of the position of the tape during treatment is also recommended to provide rest to the tissues in case they become irritated. It is also recommended that an aloe vera gel be applied to the cheeks when changing tapes.

## DISCUSSION

There are several benefits of the nasoalveolar moulding technique in the treatment of cleft lip and palate deformity. A proper alignment of the alveolus, lip and the nose helps the surgeon to achieve a better and more predictable surgical result. The cleft deformity is significantly reduced in size with the NAM therapy before surgery, making primary repair of the lip, alveolus and the nose an effortless procedure. The approximation of the alveolar processes before surgery also enables the surgeon to perform gingivoperiosteoplasty successfully. Long-term studies of NAM therapy indicate that the change in the nasal shape is stable with less scar tissue and better lip and nasal form.[19] This improvement reduces the number of surgical revisions for excessive scar tissue, oronasal fistulas, and nasal and labial deformities.[20] With the alveolar segments in a better position and increased bony bridges across the cleft, the permanent teeth have a better chance of eruption in a good position with adequate periodontal support.[21] Studies have also demonstrated that 60% of patients who underwent NAM and gingivoperiosteoplasty did not require secondary bone grafting.[22] The remaining 40% who did need bone grafts showed more bone remaining in the graft site compared to patients who have had no gingivoperiosteoplasty.[21] Fewer surgeries also result in substantial cost savings for families and insurance companies.[23] Lee *et al*. demonstrated that midfacial growth in the sagittal and vertical plane was not affected by NAM and gingivoperiosteoplasty.[24] Since the initiation of NAM, there has been a significant difference in the outcome of the primary surgical cleft repair. With proper training and clinical skills, NAM has demonstrated tremendous benefit to the cleft patients as well as to the surgeon performing the primary repair.
